# New Coleoptera records from New Brunswick, Canada: Dryopidae, Elmidae, Psephenidae, and Ptilodactylidae

**DOI:** 10.3897/zookeys.179.2604

**Published:** 2012-04-04

**Authors:** Reginald P. Webster, Ian DeMerchant

**Affiliations:** 1 Natural Resources Canada, Canadian Forest Service, Atlantic Forestry Centre, 1350 Regent St., P.O. Box 4000, Fredericton, NB, Canada E3B 5P7

**Keywords:** Dryopidae, Elmidae, Psephenidae, Ptilodactylidae, new records, Canada, New Brunswick, Maritime provinces

## Abstract

We report five new species records for New Brunswick, Canada from the Coleoptera families Dryopidae, Elimidae, Psephenidae, and Ptilodactylidae. *Dryops viennensis* (Heer) (Dryopidae) and *Promoresia elegans* (LeConte) (Elmidae) are added to the faunal list for New Brunswick and the Maritime provinces. Two Psephenidae species, *Ectopria nervosa* (Melsheimer) and *Ectopria thoracica* (Ziegler) are reported for the first time for New Brunswick, and the latter species is also new for the Maritime provinces. *Anchytarsus bicolor* (Melsheimer) and the family Ptilodactylidae are newly recorded for New Brunswick and the Maritime provinces. Collection, habitat data, and distribution maps are presented for all of these species.

## Introduction

This paper reports new records from New Brunswick, Canada of the Coleoptera families Dryopidae, Elmidae, Psephenidae, and Ptilodactylidae. There have been no recent records of these families from New Brunswick or the region since the publications of LeSage (1991a, b, c, d). Sampling in New Brunswick by the first author since 2003 has yielded additional new provincial records in the above families. The purpose of this paper is to report on these new records. A brief synopsis of each family is included in the results below.

## Methods and conventions

### Collection methods

Various methods were employed to collect the specimens reported in this study. Details are outlined in Webster et al. (2009, Appendix). Specimens in the family Ptilodactylidae were collected as by-catch in Lindgren 12-funnel traps during a study to develop improved tools for detection of invasive species of Cerambycidae. See Webster et al. (in press) for details of the methods used to deploy funnel traps and for sample collection. A description of the habitat was recorded for all specimens collected during this survey. Locality and habitat data are presented exactly as on labels for each record. This information, as well as additional collecting notes, are summarized and discussed in the collection and habitat data section for each species.

### Distribution

Distribution maps, created using ArcMap and ArcGIS, are presented for each species in New Brunswick. Every species is cited with current Distribution in Canada using the following abbreviations for the provinces. New records for New Brunswick are indicated in bold under Distribution in Canada.

Acronyms of collections examined or where specimens reside referred to in this study are as follows:

**Table:** 

**ON**	Ontario	**NS**	Nova Scotia
**QC**	Quebec	**NF & LB**	Newfoundland and Labrador
**NB**	New Brunswick		

CNCCanadian National Collection of Insects, Arachnids and Nematodes, Agriculture and Agri-Food Canada, Ottawa, Ontario, Canada


NBMNew Brunswick Museum, Saint John, New Brunswick, Canada


RWCReginald P. Webster Collection, Charters Settlement, New Brunswick, Canada


## Results

### Species accounts

All records below are species newly recorded for New Brunswick, Canada. Species followed by ** are newly recorded from the Maritime provinces (New Brunswick, Nova Scotia, Prince Edward Island) of Canada.

The classification of the Dryopidae, Elmidae, Ptilodactylidae, and Psephenidae follows Bouchard et al. (2011).

### Family Dryopidae Billberg, 1820

In North America, the Dryopidae (long-toed water beetles) are generally aquatic as adults and terrestrial as larvae; they are herbivorous (Shepard 2002b). Adults are usually found in riffle areas of the streams in leaf packs, log jams or other stream substrates. The larvae occur in moist soil along creek margins (LeSage 1991a). Five species of Dryopidae were reported from Canada by LeSage (1991a), including the adventive *Dryops viennensis* (Heer). Only *Helichus basalis* LeConte and *Helichus striatus* LeConte were reported from New Brunswick by LeSage (1991d). Here, we newly record *Dryops viennensis* from New Brunswick (Table 1).

**Table 1.** Species of Dryopidae, Elmidae, Psephenidae, and Ptilodactylidae recorded from New Brunswick, Canada.

Family Dryopidae Billberg
*Dryops viennensis* (Heer)**
*Helichus basalis* LeConte
*Helichus striatus* LeConte
Family Elmidae Curtis
Subfamily Elminae Curtis
Tribe Elmini Curtis
*Microcylloepus pusillus pusillus* (LeConte)
*Optioservus fastiditus* (LeConte)
*Optioservus ovalis* (LeConte)
*Optioservus trivittatus* (W J Brown)
*Oulimnius latiusculus* (LeConte)
*Promoresia elegans* (LeConte)**
*Promoresia tardella* (Fall)
*Stenelmis crenat* (Say)
*Stenelmis mera* Sanderson
Tribe Macronychini Gistel
*Macronychus glabratus* Say
Family Psephenidae Lacordaire
Subfamily Eubrianacinae Jakobson
*Ectopria nervosa* (Melsheimer)**
*Ectopria thoracica* (Ziegler)*
Subfamily Psepheninae Lacordaire
*Psephenus herricki* (DeKay)
Family Ptilodactylidae Laporte
Subfamily Anchytarsinae Champion
*Anchytarsus bicolor* (Melsheimer)**

Notes: *New to province, **New to Maritime provinces.

#### Dryops viennensis

(Heer, 1841)**

http://species-id.net/wiki/Dryops_viennensis

Map 1

##### Material examined.

**New Brunswick, Carleton Co.**, (Jackson Falls) “Bell Forest”, 46.2152°N, 67.7190°W, 21.VIII.2004, R. P. Webster, river margin, under cobbles (1, RWC); Hartland, Becaguimec Island (in Saint John River), 46.3106°N, 67.5372°W, 16.IX.2006, R. P. Webster, river margin, under cobbles (1, RWC); Meduxnekeag Valley Nature Preserve, 46.1888°N, 67.6762°W, 19.VII.2009, R. P. Webster, river margin, under rock (1, RWC). **Madawaska Co.**, Baker Brook, island in Saint John River, 47.2972°N, 68.5123°W, 26.VII.2006, R. Capozi & R. Webster, river margin among cobblestones near water (1, RWC); 4.0 km W of Saint-Hilaire on Saint John River, 47.2875°N, 68.4586°W, 27.VII.2006, R. Capozi & R. Webster, river margin among cobblestones near water (1, RWC). **Restigouche Co.**, confluence of Restigouche River and Stillwater Brook, 19.VIII.1999, R. Webster & D. Arseneault, rocky and gravel river margin, under cobbles (3, RWC); Jacquet River Gorge P.N.A., 47.8256°N, 66.0770°W, 13.VIII.2010, R. P. Webster, large shaded brook, among cobblestones (1, NBM).

##### Collection and habitat data.

Nearly all adults of *Dryops viennensis* from New Brunswick were found along rivers and larger brooks under or among cobblestones above the waterline but close to the edge of the water. Adults collected during July, August, and September.

##### Distribution in Canada and Alaska.

QC, **NB** (LeSage 1991d).

**Map 1.**
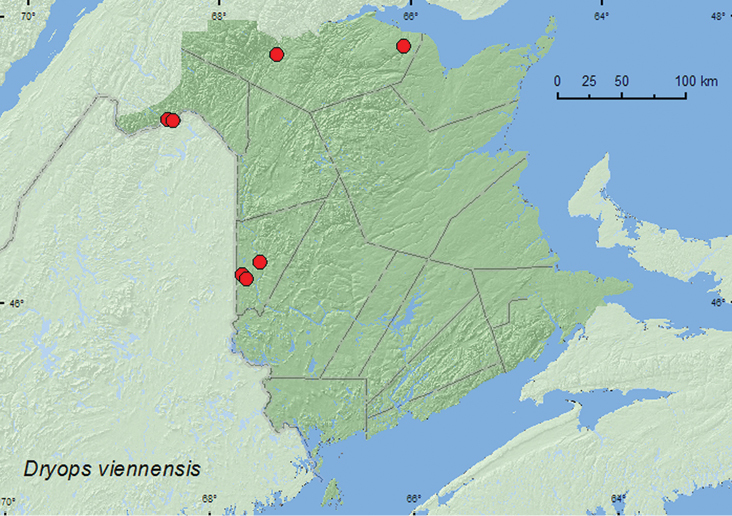
Collection localities in New Brunswick, Canada, of *Dryops viennensis*.

### Family Elmidae Curtis, 1830

The Elmidae (riffle beetles) occurring in eastern Canada are aquatic both in the larval and adult stages and seldom leave the water (LeSage 1991b; Shepard 2002a). Most elmids live in cool, rapid-flowing, and well-oxygenated streams, and adults and larvae feed on diatoms, encrusted algae detritus, or submerged decaying wood (LeSage and Harper 1976a; Shepard 2002a). Thirty-two species were reported from Canada, including ten species from New Brunswick (LeSage 1991b). Here, we report an additional species for the province (Table 1).

### Subfamily Eliminae Curtis, 1830

#### Promoresia elegans

(LeConte, 1852)

http://species-id.net/wiki/Promoresia_elegans

Map 2

##### Material examined.

**New Brunswick, Carleton Co.**, Jackson Falls, Bell Forest, 46.2208°N, 67.7211°W, 28.VI.2005, R. P. Webster, mature hardwood forest, u.v. light (1, RWC).

##### Collection and habitat data.

The single adult was captured during late June at an ultraviolet light deployed in a mature hardwood forest. A small, cold, spring-fed brook occurred adjacent to the site where the light was set up.

##### Distribution in Canada and Alaska. 

QC, **NB** (LeSage 1991a).

**Map 2.**
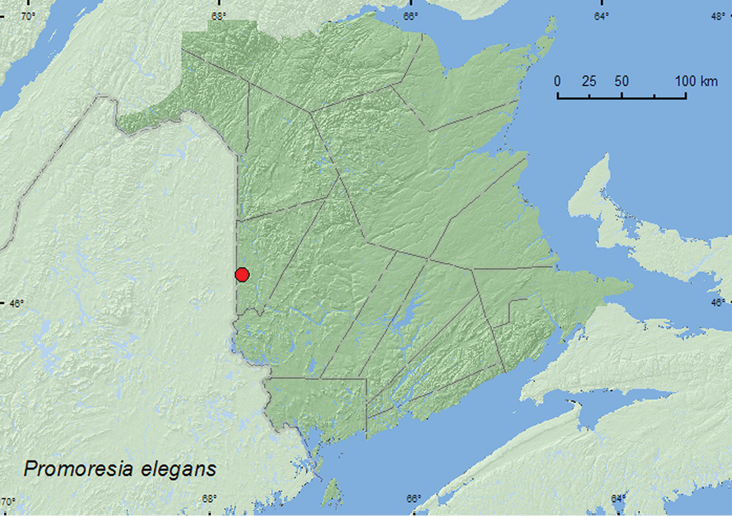
Collection localities in New Brunswick, Canada, of *Promoresia elegans*.

### Family Psephenidae Lacordaire, 1854

The Psephenidae (the water penny beetles) is a small family of riparian species associated with brooks, streams, and rivers. The larvae, which are aquatic, are usually found on stones or on submerged wood in fast-flowing water (Shepard 2002c). The larvae of *Psephenu*s are flat and disk shaped, and are found on rocks in streams, and thus their common name water penny beetles (Shepard 2002c). Adults are found on rocks or foliage near streams and are often attracted to lights. Three species of Psephenidae were reported from Canada by LeSage (1991c). *Psephenus herricki* (DeKay) was the only species recorded from New Brunswick. *Ectopria thoracica* was treated as a synonym of *Ectopria nervosa* by LeSage (1991c). However, Brigham (1981) treated *Ectopria nervosa* and *Ectopria thoracica* as distinct species, based on differences in genitalia and coloration, and provided a key to separate members of the genus. We treat them as two distinct species, and both are newly recorded from New Brunswick (Table 1).

### Subfamily Eubrianacinae Jakobson, 1913

#### Ectopria nervosa

(Melsheimer, 1845)

http://species-id.net/wiki/Ectopria_nervosa

Map 3

##### Material examined.

**New Brunswick, Carleton Co.**, (Jackson Falls) Bell Forest, 46.2208°N, 67.7211°W, 13.VII.2004, 28.VI.2005, R. P. Webster, mature hardwood forest, u.v. light (4, RWC); Meduxnekeag Valley Nature Preserve, 46.1888°N, 67.6762°W, 4.VII.2005, R. P. Webster, river margin, sweeping foliage (2, RWC); same locality and collector but 46.1957°N, 67.6803°W, 28.VI.2005, mixed forest, u.v. light trap (3, RWC).

##### Collection and habitat data.

*Ectopria nervosa* was collected at an ultraviolet light and in an ultraviolet light trap deployed in a mature hardwood forest and a mixed forest. Small spring-fed brooks were in the vicinity of the sites where the lights were used. Two individuals were swept from foliage along a river margin (clear, fast-flowing, rocky river). Adults were captured during June and July in New Brunswick.

##### Distribution in Canada and Alaska.

ON, QC, **NB**, NS (Downie and Arnett 1996; LeSage 1991c). There are specimens from Ontario and Quebec in the CNC (Laurent LeSage, personal communication).

**Map 3.**
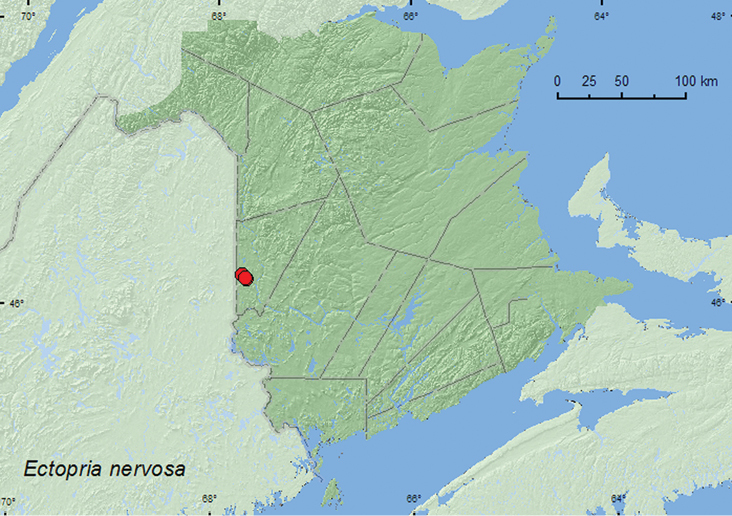
Collection localities in New Brunswick, Canada, of *Ectopria nervosa*.

#### Ectopria thoracica

(Ziegler, 1845)**

http://species-id.net/wiki/Ectopria_thoracica

Map 4

##### Material examined.

**New Brunswick, York Co.**, Charters Settlement, 45.8395°N, 66.7391°W, 17.VII.2004, 27.VII.2004, 4.VII.2005, 29.VI.2006, 27.VI.2007, R. P. Webster, mixed forest, u.v. light (9, RWC); same locality and collector but 45.8430°N, 66.7275°W, 11.VII.2005, regenerating forest, beating foliage (1, RWC).

##### Collection and habitat data.

Adults of this species were captured at an ultraviolet light deployed adjacent to a mixed forest with a nearby medium-sized, clear, rocky stream. Adults were captured during June and July.

##### Distribution in Canada and Alaska.

ON, QC, **NB** (Downie and Arnett 1996). There are specimens from Ontario and Quebec in the CNC (Laurent LeSage, personal communication).

**Map 4.**
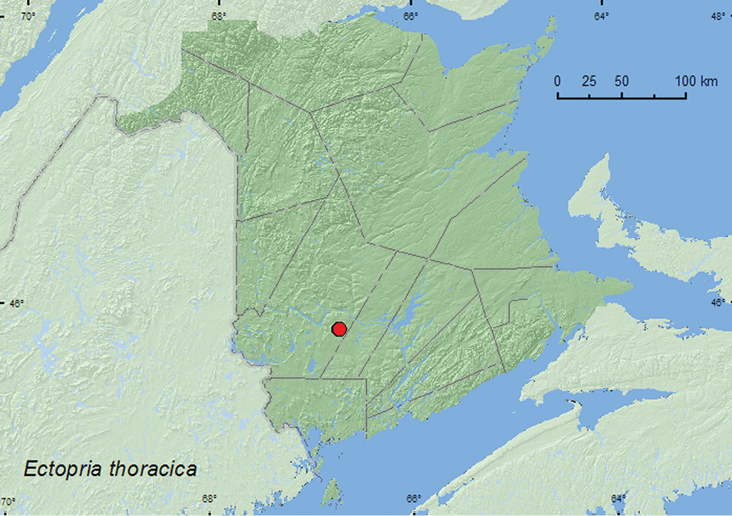
Collection localities in New Brunswick, Canada, of *Ectopria thoracica*.

### Family Ptilodactylidae Laporte, 1836

The Ptilodactylidae (ptilodactylid or toed-winged beetles) are primarily tropical in distribution and only three species are known from Canada (LeSage 1991d). Depending on the species, larvae occur in and feed on decaying vegetation in aquatic or damp terrestrial habitats (Ivie 2002; LeSage and Harper 1976b). Adults are taken at lights or beaten from vegetation, usually near riparian habitats (LeSage 1991d; Ivie 2002). Adult Ptilodactylinae feed on spores (Stribling and Seymour 1988), otherwise little is known about the feeding habits of other groups. No species of Ptilodactylidae were reported from New Brunswick by LeSage (1991d). Here, we report *Anchytarsus bicolor* (Melsheimer) and the family Ptilodactylidae for the first time for New Brunswick and the Maritime provinces (Table 1.).

### Subfamily Anchytarsinae Champion, 1897

#### Anchytarsus bicolor

(Melsheimer, 1846)**

http://species-id.net/wiki/Anchytarsus_bicolor

Map 5

##### Material examined.

**New Brunswick,** **Charlotte Co.**, 10 km NW of New River Beach, 45.2110°N, 66.6170°W, 29.VI-16.VII.2010, R. Webster & C. MacKay, old growth eastern white cedar forest, Lindgren funnel traps (5, CNC, RWC).

##### Collection and habitat data.

Larvae of *Anchytarsus bicolor* feed on rotten wood of submerged, water-logged logs in slow-flowing streams (LeSage and Harper 1976b; Stribling 1986). Adults of this uncommon species have been collected at lights and from under leaf litter along stream margins (LeSage and Harper 1976b). Specimens from New Brunswick were captured in Lindgren funnel traps deployed near a slow-flowing stream in an old-growth eastern white cedar (*Thuja occidentalis* L.) swamp. Adults were captured between late June and mid July. Elsewhere, this species has been collected from May to July (Stribling 1986).

##### Distribution in Canada and Alaska.

ON, QC, **NB** (LeSage and Harper 1976b; LeSage 1991d)

**Map 5.**
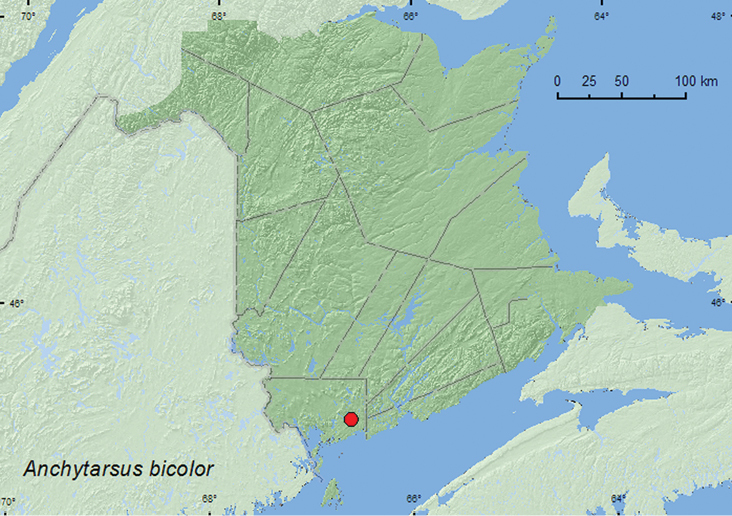
Collection localities in New Brunswick, Canada, of *Anchytarsus bicolor*.

## Supplementary Material

XML Treatment for Dryops viennensis

XML Treatment for Promoresia elegans

XML Treatment for Ectopria nervosa

XML Treatment for Ectopria thoracica

XML Treatment for Anchytarsus bicolor
